# Retrospect

**Published:** 1889-02

**Authors:** 


### Editorial.

### RETROSPECT.

The present number completes the Fifth Volume of the New Series of the JOURNAL. It may not be inappropriate to pause at this point, and looking back over the past year to consider the work which the JOURNAL is doing for its readers. Our innate modesty forbids an indulgence in self-laudation, yet we cannot refrain from congratulating ourselves upon the degree of success with which we have justified the claim that we give to our subscribers the best Medical Journal in the South. Without giving undue prominence to any one department, our pages have presented a valuable array of original articles of undoubted merit, of reports of the proceedings of some of the most active and progressive medical societies in the United States, extracts and translations of the latest and best material from American and foreign medical literature, and editorial articles which must speak for themselves. In short, we have furnished a journal which enables the busy practitioner to keep abreast with the progress of his art, to know what is going on in the medical world, and to extend the range of his professional vision beyond the humdrum round of daily practice. No medical journal can take the place of systematic reading of standard works on medicine, and the JOURNAL has not this aim in view. To make the attempt would expose it to the fate which awaits every misdirected effort. Yet in its own sphere the one is no less important than the other, and to properly fill this sphere is all that we seek to do. How well we have succeeded in accomplishing this purpose during the past year, is a question which our readers must decide for themselves.

No medical journal prospers by the unaided efforts of its editorial management. It must depend to a large extent upon the efforts of those of its readers who contribute to its pages the fruits of their own experience and observation. In this direction the JOURNAL owes a debt of gratitude, which it is a pleasure to publicly acknowledge. Especially are our thanks due for the efficient assistance rendered in the editorial department by Prof. Hunter P. Cooper, of Atlanta, whose graceful and facile pen has often lent its aid in the service of our readers.

If in some respects the JOURNAL has fallen short of the ideal at which it aims, it is because the standard which it has set for itself is a lofty one. Realizing fully the fact that good intentions are valueless, except as they yield a fruition of good results, we close the present volume, relying upon the judgment of our readers for an honest verdict upon the merits of our past year's work.

### ITEMS.

THE INDEX to volume V will be issued with the March number.

A PARTNERSHIP has been formed by Drs. Joseph P. Logan and W. S. Kendrick, for the practice of medicine and surgery.

DR. WM. A. HAMMONDS' Sanitarium was opened for the reception of patients on January 6th, when more than half of its rooms were filled.

DR. W. H. WAY, lately House Surgeon in the Manhattan Eye and Ear Hospital, New York, has located in Austin, Texas. We commend Dr. Way to our Texas friends as a gentleman, and one who is thoroughly posted in his chosen specialty.

MESSRS. J. B. LIPPINCOTT COMPANY to announce the profession the publication of a "Cyclopedia of the Diseases of Children," medical and surgical, by American, British and Canadian authors, edited by John M. Keating, M. D., in four imperial octavo volumes; to be sold by subscription only. The first volume will be issued early in April, and the subsequent volumes at short intervals.

A thorough knowledge of the diseases of children is a matter of the greatest importance to most physicians, and as this is the only work of the kind that has been published in English, it will be invaluable as a text-book and work of reference for the busy practitioner.

E. B. TREAT, Publisher, 771 Broadway, New York, will publish, early in 1889, the Seventh Annual Issue of the English "Medical Annual," a *resume* in dictionary form, of New Remedies and New Treatment that have come to the knowledge of the profession throughout the world during 1888. The editorial staff of the forthcomig volume will include articles or departments edited by Sir Morrell McKenzie, M. D. (Laryngology), London; Jonathan Hutchinson, Jr., M. D. (Genito-Urinary Diseases), London; J. W. Taylor, M. D. (Gynecology), Birmingham; William Lang, M. D. (Ophthalmologist), London; James R. Leaming, M. D. (Heart and Lung), New York; Charles L. Dana, M. D. (Neurologist), New York; H. D. Chapin, M. D. (Pediatrics), New York; and others, comprising a list of twenty-three collaborators, widely known in Europe and America. n its enlarged and widened sphere it will take the name of "The International Medical Annual," and will be published in one octavo volume of about 600 *pages at* $2.75, and issued simultaneously in London and New York.

SHOULD A DOCTOR INSURE HIS LIFE ?—There is something startling in the almost daily announcement that Dr. A. or Dr. B.

has died, after practicing medicine these many years, and has left nothing for the adequate support of his family. It matters little whether the doctor's practice has been small or large, at death nearly all have one thing in common, viz., poverty.

There is one way by which this could be avoided with certainty, viz., by insuring one's life.

It is a common principle with business men to insure their material possessions, that afford them a regular income. Hence, if fire or other disaster overtake these possessions the insurance will make good part or all the loss.

In the case of the doctor, his material possessions are his health, his brains, his skill, etc. This that is wrapped up in his body brings him an income varying from five hundred dollars to fifty or sixty thousand dollars per year. If his body dies this income stops at once. Is it not in accord with business principles to insure this body that is worth so much in cash, for a portion if not all the capital which, invested at usual rates of interest, would produce a sum approaching the yearly earnings of the doctor? Were this done, then at his death the doctor's family would have a portion if not all of the income that the doctor earned while alive. Hence they could continue to live much as they did during his active life.

We are convinced that this matter is insufficiently considered by the great mass of doctors. Some carry insurance as large as their incomes will admit, but relatively few.

Of all men, the doctor is most familiar with the uncertainty of life, and the certainty of death. He is also familiar with the distress occasioned by the failure of the head of a family to make adequate provision for those dependent upon him.

There are few physicians that can spare time from the duties of active practice to engage in other pursuits from which they might accumulate a competence for their families. The practice of medicine is a very exacting calling. Something could be yearly invested in life insurance, and this amount could be increased as the yearly income became larger.

It is objected that some insurance companies fail. The reply is, certainly, but there are insurance companies that are less likely to fail than the United States is to be dissolved.

He who desires to place his life insurance in safe hands can do so with more certainty than he can deposit money in a safe bank.

Every doctor who has not an independent fortune, should possess some life insurance, an amount proportioned to his ability to pay the annual premiums without embarrassment. Thus he will at once provide his family against the possibility of their suffering absolute want in case of his sudden death. Thus he will be gradually accumulating a fund upon which he can draw himself should he become disabled or suffer reverses, or need during the years of a declining life.

It is a good time to begin the year by making a start in this sort of provision for the future.—*American Lancet.*

THE *N. Y. Med. Abstract*, Nov., 1888, says that M. Gariel, a professor in the Nantes Medical School, proposes a combination of two important therapeutic measures in his method of "general hydropathic electrization." This consists simply of a shower bath to which wires are attached in such a way as to pass electric currents through the water at the time of using it. Patients who have been unable to take the ordinary cold douche appear to have used this modification of it with advantage, on account of the quick reaction which follows its employment.

